# Comparison of different settings for yellow subthreshold laser treatment in diabetic macular edema

**DOI:** 10.1186/s12886-018-0841-z

**Published:** 2018-07-11

**Authors:** Jay Chhablani, Rayan Alshareef, David Ta Kim, Raja Narayanan, Abhilash Goud, Annie Mathai

**Affiliations:** 1 Smt. Kanuri Santhamma Retina Vitreous Centre, L.V.Prasad Eye Institute, Kallam Anji Reddy Campus Banjara Hills, Hyderabad, 500034 India; 2 Department of Ophthalmology, McGill University, Montreal, Quebec Canada; 3 Department of Surgery, Division of Ophthalmology, Faculty of Medicine and Health Sciences, Université de Sherbrooke, Sherbrooke, Quebec Canada

**Keywords:** Subthreshold laser, Microsecond, Micropulse, Diabetic macular edema, Microperimetry, Laser photocoagulation

## Abstract

**Background:**

To assess the safety and efficacy of two subthreshold parameters (5 and 15% duty cycle (DC)) compared to standard ETDRS (early treatment of diabetic retinopathy study) continuous wave (CW) laser.

**Methods:**

In this prospective randomized study, 30 eyes from 20 patients with non-center involving macular edema were randomized into 3 different groups: 5% DC, 15% DC and CW navigated modified ETDRS laser treatment. Titration in subthreshold groups was performed with 30% of the threshold power, decided with microsecond pulses. CW laser was titrated to a barely visible burn. All patients underwent microperimetry, thickness measurements and visual acuity examinations at baseline, 6 weeks and 12 weeks post treatment.

**Results:**

At three months follow up, retinal sensitivity was significantly reduced in the CW group by − 2.2 dB whereas in both subthreshold groups, retinal sensitivity increased by 2.4 dB for 5% and 1.9 dB for 15% DC with no significant difference. Retinal volume (mm^3^) decreased in both subthreshold groups by 0.08 ± 0.3 and 0.12 ± 0.11 in 5 and 15% DC group respectively. Whereas the CW group showed volume increase of 0.55 ± 0.92 (*p* = 0.02 and 0.01 for 5 and 15% DC groups). Visual acuity remained stable in all 3 groups (− 0.7 letter in 5% DC; 2.11 letters in 15% DC and 0.88 in CW with no significant difference).

**Conclusion:**

Subthreshold microsecond laser was shown to be safe and effective with both 5 and 15% DC as compared to conventional photocoagulation with ETDRS parameters. The 15% DC setting trended to achieve better anatomical, visual and functional outcomes.

**Trial registration:**

Retrospectively registered (NCT03571659, 06/26/2018).

## Background

After various randomized and non-randomized clinical trials, anti- vascular endothelial growth factor (VEGF) therapy has become a gold standard in management of diabetic macular edema [1–5]. However, in the RISE and RIDE trials, the Phase 3 trials for ranibizumab in diabetic macular edema, 13.9% of patients receiving monthly intravitreal injections of ranibizumab showed no gain of letters compared to baseline at 24 months [3]. Frequent visits with frequent injections, causes a major economic burden for these patients. In a study from Wallick et al., patients with diabetic macular edema were found to have, on average, 25.5 annual days with a health care related visit [6]. In a Canadian study published in 2014 by Gonder et al., the mean 6-month DME-related cost was $2092 per patient [7]. The present day cost is likely higher since the study considered that 70% of patients were injected with bevavizumab and the rest with ranibizumab. Thus, there is a constant need for a therapy with long term efficacy for this visually debilitating disease.

Laser photocoagulation was proposed as treatment of choice for diabetic macular edema after ETDRS (Early Treatment of Diabetic Retinopathy Study) [8], much before the anti-VEGF era. Maintenance rather than the vision improvement along with the loss of contrast sensitivity, poor color vision, accidental foveal damage and expansion of macular scars were the primary complication of laser photocoagulation, which brought the laser photocoagulation in the back seat. However, in recent past with improved technology, subthreshold laser photocoagulation has got more interest and shown to be effective in various macular diseases such as diabetic macular edema [9–13], CSCR (central serous chorioretinopathy) [14–16], and venous occlusions [17].

Laser parameters were standardized after ETDRS study for diabetic macular edema [15]. Unlike conventional laser photocoagulation, there is no standard parameters have been proposed for subthreshold laser. In the literature, there is a lot of variability for subthreshold laser settings in terms of titration, duty cycle (ranging from 5 to 15% duty cycle (DC)), laser power and pulse duration.

This study aims to assess the safety and efficacy of two of the most frequently used subthreshold parameters (5 and 15% DC) when compared to standard ETDRS threshold laser.

## Methods

This prospective randomized double-masked pilot study was performed at L V Prasad eye institute, Hyderabad. The study was approved by the institutional review committee and adhered to the tenets of the Declaration of Helsinki. All participants gave written informed consent before enrollment in the study. The Hyderabad Eye Research Foundation, India, supported this study. Patients were recruited from January 2012 through February 2013 at LV Prasad Eye Institute, Hyderabad, India.

### Patient eligibility

Inclusion criteria: Eyes with naive non-center involving macular edema (central subfield thickness less than 350 μm) with visual acuity 20/30 or better. The key exclusion criteria were: (1) Dense lens opacity impeding the visualization or laser photocoagulation; (2) Previous macular laser photocoagulation in the study eye; (3) Use of intraocular or periocular corticosteroids in the study eye within the previous 3 months; (4) Previous treatment with anti-VEGF drugs in the study eye.

### Study design

Subjects were randomized into 3 different groups. Groups A and B received navigated microsecond laser treatments at 5% DC (100 μs on time) and 15% DC (300 μs on time), respectively. Group C received a continuous wave (CW) navigated ETDRS threshold laser treatment with visible endpoints. In situation when both eyes of the patient were eligible, each eye was randomized as per the randomization. Patient and the visual acuity assessor were masked.

### Color fundus photograph

Color fundus photographs of the optic disc, macula, and temporal retina (30°) were captured with a mydriatic camera (Zeiss FF450, Carl Zeiss Meditec, Jena, Germany).

### Fundus fluorescein angiography (FFA)

FFA was performed using fluorescein sodium 20% and imaging on Navilas® system (OD-OS GmbH, Teltow, Germany) to determine the site of leakage at baseline, and at three months from baseline.

Spectral Domain Optical Coherence Tomography (SD-OCT):

Cirrus HD-OCT (Carl Zeiss Meditec, Inc., Dublin, CA.) was used to obtain SD-OCT scans. Scanning protocol included HD5 line raster, HD single line raster, enhanced depth imaging, and macular cube. Central retinal thickness (CRT) (1 mm central retinal thickness area as described in the Early Treatment Diabetics Retinopathy Study (ETDRS) fields) was determined automatically and analyzed by OCT software, by generating images using the Macular Cube 512 × 128 scan over 6 × 6 mm area, the cube being composed of 128 horizontal examination lines of 512 A-scans each.

### Microperimetry (MAIA ™, Centervue, Padova, Italy)

Microperimetry was performed using the microperimeter (MAIA ™, Centervue, Padova, Italy) after dilation of pupils. Goldmann III stimuli and a 4–2-1 staircase strategy with a test grid with 37 stimulus locations covering an area of 10 degrees was applied. Fixation was tracked using built-in fixation target. The stimuli were projected on a white background with black illumination set to 1.27 cd/m2 and a stimulus presentation time of 200 milliseconds. Mean differential light sensitivity in decibels (dB) of all test locations was analyzed for the study. Three fixation classes were defined: stable, relatively unstable, unstable. Stable if more than 75% of the fixation points were inside the 2 degree diameter circle; relatively unstable if less than 75% were inside the 2 degree diameter circle, but more than 75% inside the 4 degree diameter circle; and unstable if less than 75% of the fixation sites were inside the 4 degree diameter circle. A change in sensitivity of 1 dB or more, a change in stability of fixation, or both was considered significant.

All patients underwent microperimetry, thickness measurements and visual acuity examinations at baseline, 6 weeks and 12 weeks post treatment.

### Laser photocoagulation

Subthreshold laser used was 577 nm navigated laser using Navilas® system (OD-OS GmbH, Teltow, Germany), however, conventional laser was 532 nm using PASCAL® (OptiMedica) system. Both subthreshold laser groups (A and B) were treated with confluent grids to cover areas of diffuse edema whereas the threshold laser group was treated with mETDRS modified grids. Fluorescein angiography was used to identify and target leaking microaneurysms, which were targeted directly using navigated laser in all groups.

Titration and settings: Titration in subthreshold groups (5 and 15% DC group) was performed with microsecond pulses to a barely visible burn, after which power was reduced to 30% to reliably achieve subthreshold effects. CW laser was titrated to a barely visible burn. Spot size and envelop pulse durations was set to 100 μm and 100 ms for each group.

#### Study visits

Patients were followed at week 6 and week 12 after the baseline visit. Comprehensive examination including microperimetry and SD-OCT were performed at all visits, however, FFA was performed at baseline and at week 12.

### Outcome measures

Primary outcome measures included change in retinal sensitivity at week 12 compared to baseline. Secondary outcome measures included change in CMT, BCVA, retinal volume on SD-OCT macular thickness map.

## Statistical analysis

Intention to treat analysis was performed. The changes compared to baseline, in retinal sensitivity, BCVA, CMT, and retinal volume, at 6 weeks and 12 weeks, were analyzed with Kruskal-Wallis-Test. *P*-value of < 0.05 was considered as statistically significant.

## Results

Thirty eyes of 20 patients with a mean age of 57 ± 8.7 years were enrolled in the study with 10 eyes in each group. Sixteen were males and four were females. At presentation, lens status was clear in 14 eyes; grade 1 nuclear sclerosis 10 and grade 2 nuclear sclerosis in 6 eyes. Baseline clinical characteristics of the groups are shown in Table 1. There was no significant difference between the groups for BCVA, CMT and retinal sensitivity.

**Table 1:** Baseline Characteristics of study groups

	Group A (5% DC)	Group B (15% DC)	Group C (CW)
Number of eyes	10	10	10
Mean duration of diabetes (years)	6.5 ± 1.3	7.1 ± 1.1	6.3 ± 2.1
Mean age (years)	58 ± 6.6	59 ± 6	57 ± 10.6
Lens status	Clear (5), NS1(2), NS2 (3)	Clear (5), NS1(4), NS2 (1)	Clear (4), NS1(4), NS2 (2)
Mean BCVA (ETDRS letters)	76 ± 10	80 ± 5	80 ± 7
Mean CMT (microns)	258 ± 28	255 ± 58	248 ± 37
Retinal Sensitivity (dB)	19 ± 5	22 ± 4	23 ± 4

*BCVA* Best corrected visual acuity

*CMT* Central Macular Thickness

**Fig. 1:**
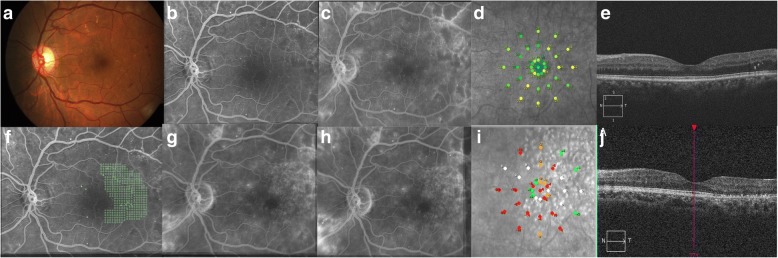
Continuous wave laser photocoagulation: Color fundus photograph (**a**) shows signs of extrafoveal macular edema with few microaneurysms in early phase of fluorescein angiogrpahy (FA) (**b**) with late leakage in late phase (**c**). Microperimetry map (**d**) shows retinal sensitivity map at baseline. Spectral domain optical coherence tomography (SD-OCT) (**e**) shows normal foveal contour with minimal extrafoveal edema. Laser planning map (**f**) on NAVILAS® device with continuous wave with 60mw power, 100 msec pulse duration, and single burn width apart. At three months follow up FA early and late phases show decrease in overall leakage along with laser scars (**g** and **h**). Microperimetry shows decrease in retinal sensitivity (**i**). SD-OCT shows normal foveal contour with outer retinal damage due to laser scars

**Fig. 2:**
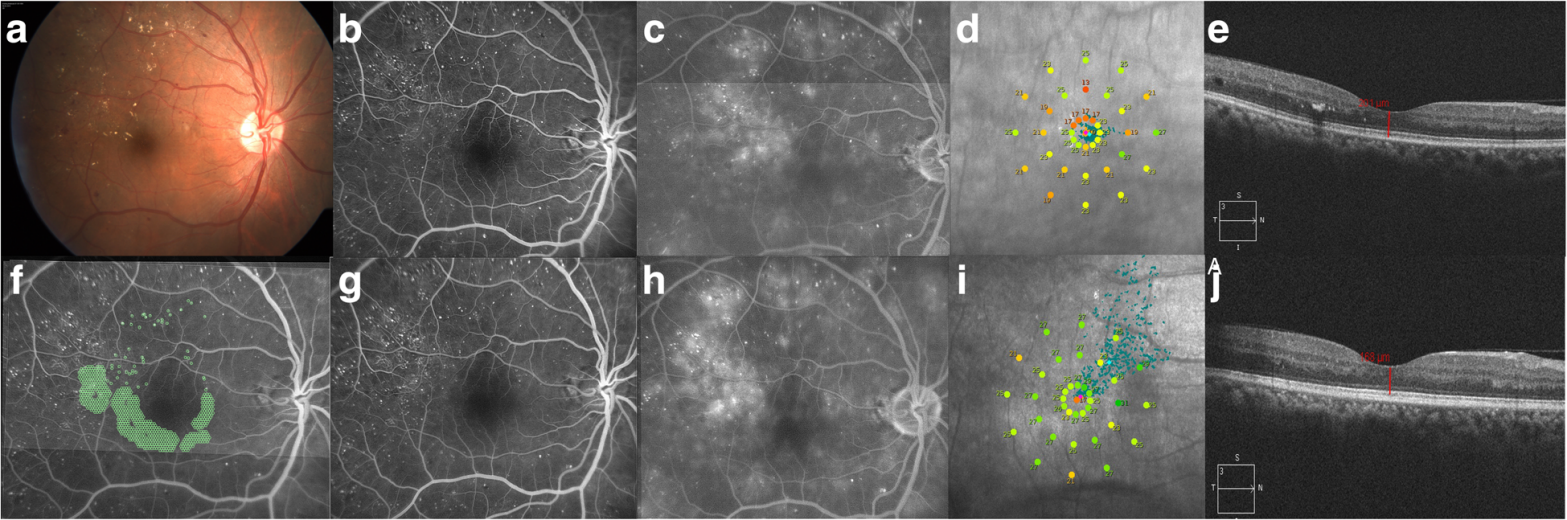
Subthreshold 5% duty cycle laser photocoagulation: Color fundus photograph (**a**) shows signs of extrafoveal macular edema with few microaneurysms in early phase of fluorescein angiogrpahy (FA) (**b**) with late leakage in late phase (**c**). Microperimetry map (**d**) shows retinal sensitivity map at baseline. Spectral domain optical coherence tomography (SD-OCT) (**e**) shows normal foveal contour with minimal extrafoveal edema. Laser planning map (**f**) on NAVILAS® device with 5%DC with 400mw power, 100 msec pulse duration, and confluent burns. At three months follow up, FA early and late phases show almost same leakage, compared to baseline without any visibile laser scars (**g** and **h**). Microperimetry shows improvement in retinal sensitivity (**i**). SD-OCT shows normal foveal contour without any outer retinal damage

**Fig. 3:**
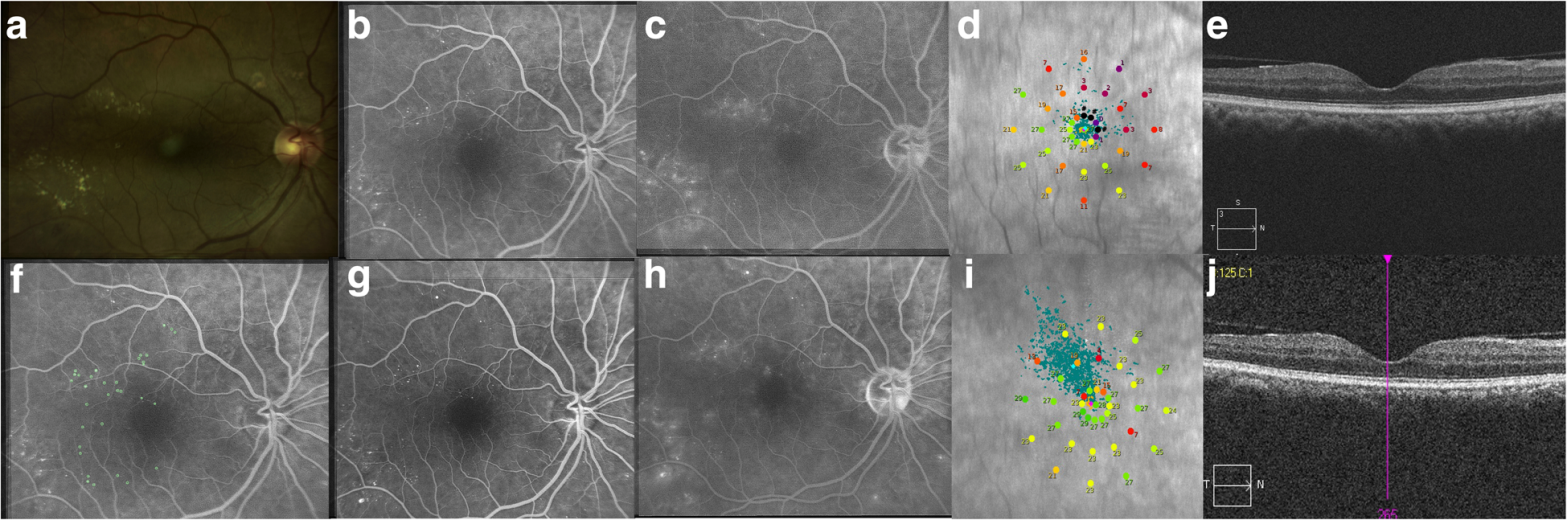
Subthreshold 15% duty cycle laser photocoagulation: Color fundus photograph (**a**) shows signs of extrafoveal macular edema with few microaneurysms in early phase of fluorescein angiogrpahy (FA) (**b**) with late leakage in late phase (**c**). Microperimetry map (**d**) shows retinal sensitivity map at baseline. Spectral domain optical coherence tomography (SD-OCT) (**e**) shows normal foveal contour. Laser planning map (**f**) on NAVILAS® device with 15%DC with 240mw power, 100 msec pulse duration, and single burn width apart. At three months follow up, FA early and late phase shows almost same leakage, compared to baseline without any visibile laser scars (**g** and **h**). Microperimetry shows improvement in retinal sensitivity (**i**). SD-OCT shows normal foveal contour without any outer retinal damage

The laser parameter used for each group is listed in Table 2. The number of spots applied was significantly lower in group C (CW) as CW laser for modified grid was one burn width apart unlike subthreshold group where confluent laser applications were performed. Representative cases are shown as Figs. 1, 2, and 3. As expected the adjusted laser power values were significantly higher in the 5%DC group as compared to 15%DC and CW. This can be attributed to the titration paradigm as the power has to be increased in order to obtain a barely visible burn to compensate the fact of a chopped microsecond pulsing laser beam. Nevertheless, the fluence in both subthreshold groups is comparable and represents about 30% of the threshold energy obtained with CW lasers.

**Table 2:** Laser parameter characteristics among different groups

	Group A (5% DC)	Group B (15% DC)	Group C (CW)
Number of spots applied	435 ± 282	335 ± 313	128 ± 112
Fluence applied mJ/mm^2^	144.4 ± 28.8	167.8 ± 28.7	488.2 ± 142.4
Power range used in mW (range)	424 ± 92.8 (220–500)	168 ± 42.9 (130–280)	87.3 ± 37.2 (50–180)

No complications were reported in any of the groups except in one eye of the 15% DC group, the evidence of the microsecond laser was detected at three months follow up on fluorescein angiography. This patient was therefore excluded from further evaluation.

### Outcome measures

Changes in primary and secondary outcome measures are shown as Table 3.

**Table 3:** Change in outcome measures at week 12

Parameters	CW	5%	15%
Retinal sensitivity (dB)	−2.2 ± 2.4	+ 2.4 ± 6.04(p*=0.3)	+ 1.9 ± 4.1(p*=0.2) Compared to 5% (*p* = 0.8)
ETDRS letters loss/gain	0.9 ± 2.5	−0.7 ± 7.7(p*=0.6)	2.11 ± 2.5 (p*=0.3) Compared to 5% (*p* = 0.3)
Central Retinal Thickness (microns)	12.3 ± 41.2	−12.4 ± 36.6 (p*=0.2)	0.6 ± 21.3(p*=0.5) Compared to 5% (*p* = 0.4)
Retinal volume (mm^3^)	+ 0.55 ± 0.92	−0.08 ± 0.3 (p*=0.02)	−0.12 ± 0.11 (p*=0.01) Compared to 5% (*p* = 0.4)

*ETDRS* Early Treatment Of Diabetic Retinopathy Study

^*^ = Compared to CW (conventional) threshold laser

#### Retinal sensitivity outcome

At three months, the retinal sensitivity was slightly reduced in the CW group by − 0.3 dB whereas in both subthreshold groups, retinal sensitivity increased by 0.9 dB for 5% and 1.7 dB for 15% DC (*p* = 0.6 and 0.2 as compared to threshold group) from baseline.

#### Visual acuity outcome

**Fig. 4:**
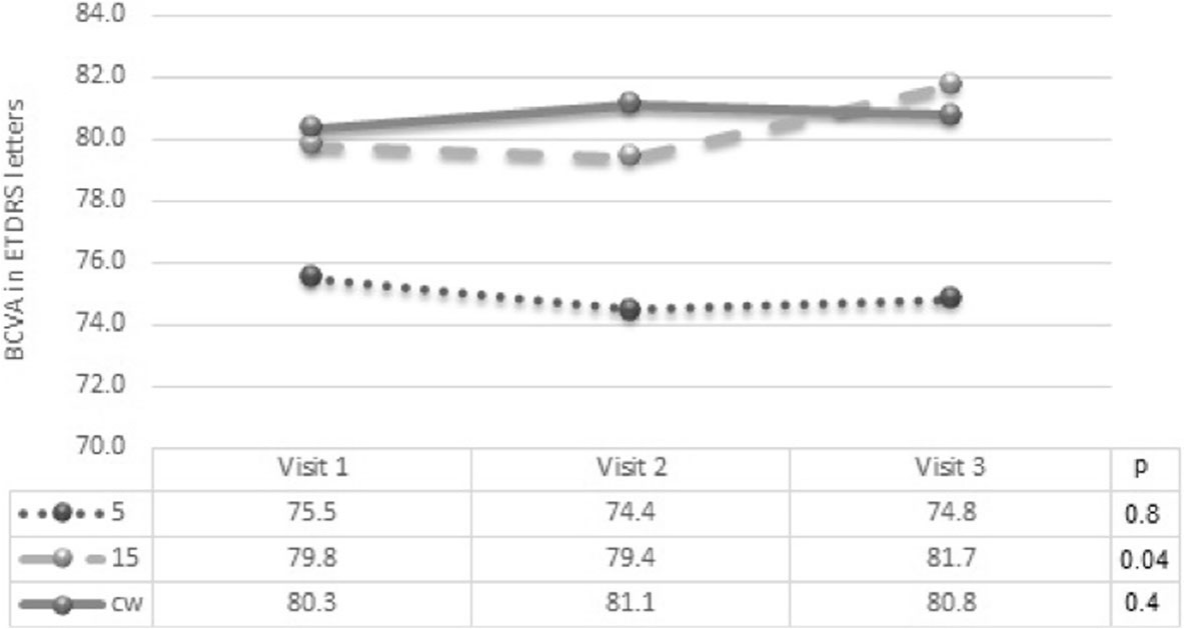
Change in best-corrected visual acuity (BCVA) during study visits

Best corrected visual acuity remained stable during the follow up period in all 3 groups with no significant difference among the groups (0.7 letter losses in 5% DC; 1.9 letters gain in 15% DC and 0.5 letters gain in CW, respectively). As indicated in Fig. 4 the 15% DC group demonstrated an improvement (*p* = 0.04). Change in BCVA is shown as Fig. 4.

#### Anatomical outcome

**Fig. 5:**
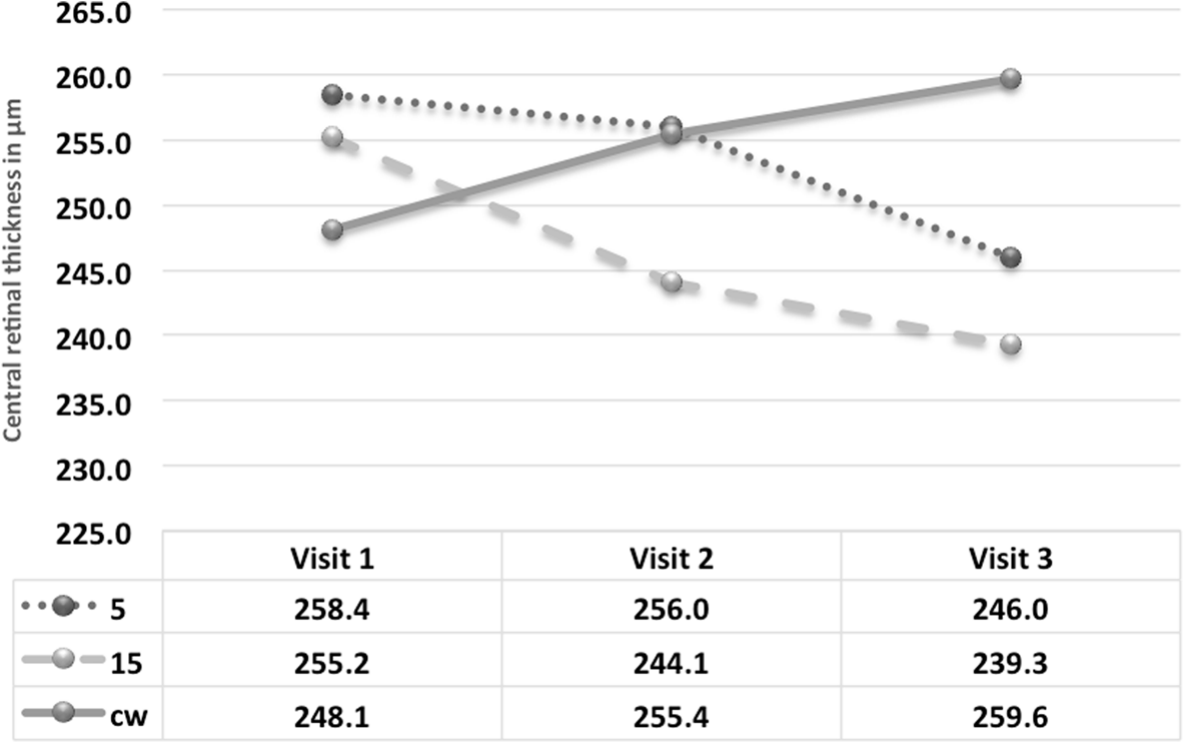
Change in central retinal thickness during study visits

Retinal volume and central retinal thickness remained stable at three months follow up, with a slight trend toward decreasing in both subthreshold groups with 0.08 ± 0.3 in 5% DC group and 0.12 ± 0.11 in 15% DC group. Whereas, CW group represented a slight volume increase of 0.55 ± 0.92 (*p* = 0.02 and 0.01 for 5 and 15% DC groups as compared to threshold group). The same applies to the CRT where a positive development of reduced CRT can be noted as compared to an increase in CRT in the CW group (See Fig. 5).

## Discussion

Subthreshold microsecond laser is a novel, tissue-sparing approach to treat diabetic macular edema. Unlike with conventional focal laser, there is no standard protocol for laser settings for subthreshold treatments. Tables 4 and 5 shows an overview of studies on subthreshold laser (including 810 nm and 577 nm) and the myriad of parameters used in diabetic macular edema [9–12, 18–26]. Vujosevic et al. compared yellow with infrared subthreshold laser in 26 and 27 eyes respectively, and found no differences in central retinal thickness, macular volume, foveal choroidal thickness, and best-corrected visual acuity [23]. Our study shows that the subthreshold microsecond laser was safe and effective with both 5 and 15% DC following careful titration as compared to CW laser. In trend, 15% DC setting seems to achieve highest ETDRS letter gain and largest decrease in volume.

**Table 4:** Overview on parameters used in 810 nm subthreshold laser treatments

	Wavelength (nm)	Spot size (um)	Duration (ms)	Duty Cycle	Power definition method
Laursen et al. [9]	810	125	100	5%	50% of barely visible burn
Figueira et al. [10]	810	125	300	30%	200% of barely visible burn
Lavinsky et al. [11]	810	125	300	15%	120% of barely visible burn
Vujosevic et al. [12]	810	125	200	5%	750 mW
Luttrull et al. [19]	810	125	300	5%	750 mW
Sivaprasad et al. [20]	810	125	200	5%	100% of a barely visible burn (unless more than 1200 mW in which case duty cycle was increased to 10%)
Othman et al. [21]	810	75–125	300	15%	100% barely visible burn
Inagaki et al. [22]	810	200	200	15%	200% barely visible burn

**Table 5:** Overview on parameters used in yellow (577 nm) subthreshold treatments

Author	Spot Size	ms	DC	Power	Fluence mmJ/mm^2^	Power definition method
Kwon et al. [24]	100	20	15%	140	54	Titration in cw; starting at 100 mW upwards until barely visible burn; after switch to μp power remains immediately below test burn
Vujosevic et al. [23]	100	200	5%	250	318	Fixed power setting
Yadav et al. [25]	100	200	10%	70–200	340	Titration burn in cw, until mild retinal whitening; then μs mode and Half power
Inagaki et al. [22]	200	200	15%	204	197	Test burn in cw mode with 100 ms and 200 μm; then switch to 15% DC and doubling the power which is 60% of threshold energy
Pei-pei et al. [30]	60	10	100%	32,4 J/cm^2^	324	50% of power, no switch of Pulse duration

Lavinsky et al. did a detailed analysis of retinal structures changes under certain fluence reductions and concluded that 30% of threshold energy does not create any tissue defects [27, 28]. Our parameters cannot be compared directly as Lavinsky et al. used CW mode with 7-10 ms pulse durations. However, considering that shortening the pulse duration results in lesser damage, we performed subthreshold microsecond laser with 30% of threshold laser power and found it to be successful. Therefore, these settings can be considered as safe and effective with microsecond laser.

One of the challenges with micropulse is the invisibility of laser applications, which makes it difficult to follow up the patients and re-treatment. NAVILAS® provides an additional advantage over other micropulse laser systems is that it provides the reports with treated area along with laser parameters. Application of confluent laser marks could be challenging using conventional slit lamp laser systems due to eye movement and the unavailability of eye tracking. NAVILAS® provides a computerized laser planning and eye tracking during laser application which is accurate and beneficial for subthreshold laser as the laser spots are not visible. Two studies have shown that use of the NAVILAS® results in higher accuracy of targets for photocoagulation compared to the conventional method without the navigating system [29–31]. However, previous reports suggest subthreshold laser application in the “whole posterior pole” which doesn’t require the information about the previously performed subthreshold laser applications. [32, 33]

Luttrull et al. showed increased burn risk for 810 nm subthreshold laser with more than 5%DC. [34] This risk increases with decreasing wavelength, which may have been the reason for visible burn in one out 10 eyes with 15%DC. This needs further clarification in terms of safety with larger sample size including 5%DC group. However, this study supports the safety of subthreshold laser over the CW laser.

Limitations of our study include small sample size in each group and short follow up. Our study did not scientifically analyze microaneurysm closure rate. However, this is the first study, which compares effect of different duty cycle subthreshold dosage with standard ETDRS laser dosage in diabetic macular edema. However, less number of subthreshold laser applications over a limited area of DME may be the reason for suboptimal response. Due to ethical issues, we did not include center-involving edema, which may have responded differently due to more severity and further loss of retinal sensitivity, and may have influenced the outcome measures.

## Conclusion

In conclusion, our pilot study reports the subthreshold laser with 15% DC appears to be more efficacious to reduce the retinal thickness and improve the retinal sensitivity, however, safety needs further evaluation on larger studies. Further studies are warranted to evaluate subthreshold laser in center-involving diabetic macular edema with or without anti-VEGF therapy. Subthreshold laser could be considered as cheap treatment option and finally a retinal restorative therapy without any structural and functional damage.

## Abbreviations

BCVA: Best Corrected Visual Acuity
CMT: Central Macular Thickness
CRT: Central Retinal Thickness
CSCR: Central Serous Chorioretinopathy
CW: Continuous Wave
DC: Duty Cycle
DME: Diabetic Macular Edema
ETDRS: Early Treatment Diabetic Retinopathy Study
FFA: Fundus Fluorescence Angiography
SD-OCT: Spectral Domain Ocular Coherence Tomography
VEGF: Vascular Endothelial Growth Factor
